# Application of surface-enhanced laser desorption/ionization time-of-flight mass spectrometry technology for the diagnosis of colorectal adenoma

**DOI:** 10.3892/ol.2013.1304

**Published:** 2013-04-15

**Authors:** ZHONG-YIN ZHOU, DI-DI TAO, JI-WANG CAO, HE-SHENG LUO

**Affiliations:** Department of Gastroenterology, Renmin Hospital of Wuhan University, Wuhan, Hubei 430060, P.R. China

**Keywords:** surface-enhanced laser desorption/ionization time-of-flight mass spectrometry, colorectal adenoma, biological marker

## Abstract

The aim of the present study was to identify a specific biological marker for the diagnosis of colorectal adenomas through the analysis of variations in serum protein profiling in colorectal adenoma patients. The study was conducted at the Renmin Hospital of Wuhan University (Wuhan, China) between September 2011 and May 2012. Surface-enhanced laser desorption/ionization time-of-flight mass spectrometry (SELDI-TOF-MS) was performed to compare the serum protein profiles of 50 patients with colorectal adenoma and 50 healthy individuals. The obtained protein profiles were analyzed using Biomarker Wizard software. Twenty protein peaks were identified to exhibit differences in average intensity between colorectal adenomas compared with normal controls, including peaks 8,565.84, 8,694.51 and 5,910.50 Da, in which the intensity between the patients and control individuals was significantly different. Two peaks, 8,565.84 and 8,694.51 Da, were observed to be highly expressed in the colorectal adenomas, however, expression was low in the control samples. By contrast, 5,910.50 Da expression was low in the colorectal adenomas and high in the controls. The results of the current study indicate that the three protein peaks may represent specific biomarkers for colorectal adenomas.

## Introduction

The oncogenic mechanisms associated with colorectal cancer still remain largely unknown, however, the hypothesis of an ‘adenoma-cancer sequence’ is now widely accepted (1,2). In total, >80% of all colorectal cancers are derived from adenomas, and the endoscopic resection of colorectal adenomas may lower the incidence of colorectal cancer by 76–90% (3). Therefore, the incidence of colorectal cancer may be decreased markedly by improving diagnostic and treatment methods (4). The majority of colorectal adenomas exhibit no symptoms and are commonly identified by chance during colonoscopy. At present, colonoscopy represents the most effective method for the diagnosis of colorectal adenoma (5). However, colonoscopy often causes a feeling of pressure, bloating or cramping at various times during the procedure. In addition, the diagnosis of colorectal cancer is often missed using this method. Therefore, the identification of an easy to perform screening method, associated with reduced patient suffering and a high rate of accuracy, is extremely important. Surface-enhanced laser desorption/ionization time-of-flight mass spectrometry (SELDI-TOF-MS) is a newly developed comparative proteomic technology (6,7) and a number of studies have identified a promising role for the technology in screening cancer markers (8–10). In the present study, SELDI-TOF-MS was used to analyze the serum protein fingerprint of colorectal adenoma patients and compare it with that of healthy control individuals, in order to identify specific serum protein biomarkers associated with colorectal adenoma.

## Materials and methods

### Participants

The present study was performed between September 2011 and May 2012 at the Department of Gastroenterology, Renmin Hospital of Wuhan University (Hubei, China). A total of 100 non-related individuals were analyzed. Of these, 54 (54%) were male and 46 (46%) were female. The individuals were separated into the colorectal adenoma group, consisting of 50 patients (average age, 51.3±4.5 years) who were diagnosed with colorectal adenoma using colonoscopy (all confirmed with pathology), and the control group, consisting of 50 healthy individuals (average age, 53.8±5.6 years). The groups were matched by age and gender. Patients with a history of colon surgery and malignant colon tumors were rejected. All participants were of Chinese ethnicity. The study was approved by the ethics committee of Renmin Hospital. Written informed consent was obtained from all patients.

### Patient samples and protein profiling

The 3-cyclohexylamine-1-propane sulfonic acid (CHAPS), 1,4-dithiothreitol (DDT), sodium acetate (NaAC), sinapic acid (SPA), acetonitrile (CAN) and trifluoroacetate (TFA) were purchased from Sigma-Aldrich (St. Louis, MO, USA). U9 buffer [9 M urea, 2% CHAPS and 1% DDT, (pH 9.0)], WCX buffer [50 mM NaAC (pH 4.5)], SPA saturated solution (100% saturated solution and 1% TFA), magnetic beads, Au/steel chips and SELDI-TOF-MS were all purchased from Ciphergen Biosystems, Inc. (Fremont, CA, USA).

Venous blood (5 ml) was obtained from each patient under fasting conditions in the morning, placed into a dry tube and left to stand for 4 h. Following this, the samples were centrifuged at 2,504 × g at 4°C for 10 min, then the supernatant was removed and centrifuged again at 626 × g for 10 min. Next, 10 *μ*l was removed from each sample and placed into a 1.5-ml centrifuge tube. U9 buffer (20 *μ*l) was added and the sample was centrifuged at 4°C for 30 min to degenerate the protein.

To determine the most appropriate chip for this study, various chemical chips, WCX2 (weak cation), SAX2 (strong anion) and IMAC (chelated with metallic ions), were utilized to test the samples. Following a comparison, the WCX2 chip was found to combine with the largest number of different proteins and revealed the most protein peaks with a stable fingerprint, therefore, WCX2 (weak cation) was selected for this analysis.

Magnetic beads (100 *μ*l) were added to a 200-*μ*l PCR tube and incubated on a magnetic platform for 1 min. Following this, the supernatant was eliminated. Next, 100 *μ*l WCX buffer was added to pre-activate the magnetic beads for 5 min. The procedures were then repeated once more. Following this, 10 *μ*l treated serum sample was added to the activated magnetic beads, mixed well and incubated at room temperature for 30 min and on a magnetic platform for 1 min. The supernatant was eliminated and 100 *μ*l WCX buffer was added to elude the WCX2 magnetic beads. The procedures were then repeated again once more. Next, 10 *μ*l eluent (1% TFA) was added and 1 *μ*l protein-rich eluent and 1 *μ*l SPA saturated solution were applied to the Au/steel chip. The chips were air-dried and analyzed by a chip reader.

The PBS II ProteinChip reader (Ciphergen Biosystems, Inc.) was used to analyze the Au/steel chip. The reader was calibrated daily using a standard polypeptide to ensure a system quality error value under 0.1%. The acquired raw data was converted by the computer to a protein fingerprint. Ciphergen protein chip 3.0 software (Ciphergen Biosystems, Inc.) was used to calibrate the data to ensure consistency between the ionic intensity and molecular mass. The parameter settings of the protein reader during chip analysis were as follows: laser intensity, 230; detection sensitivity, 8; range of optimized molecular weight, 3–50 kDa; and highest molecular weight, 200 kDa. In total, fifty spots were collected for each sample. The x-coordinate of the mass spectrum represented the mass-to-charge ratio and the y-coordinate represented the relative content of the protein.

### Statistical analysis

The statistical analysis was performed using the Biomarker Wizard software (Ciphergen Biosystems, Inc.). The difference in protein content with the same mass-to-charge ratio between the two groups was described with the peaks and presented as a P-value. In addition, data were processed using SPSS for Windows version 17.0 (SPSS Inc., Chicago, IL, USA). Data were compared between the two groups by t-test, and the Chi square test was used to count the data. P<0.05 was considered to indicate a statistically significant difference.

## Results

### Comparison of age and gender

No significant difference was observed in age and gender between the adenoma and normal control groups.

### Screening of protein peaks in colorectal adenoma serum

The protein molecular weight was set between 1,500 and 20,000 Da and a protein peak lower than 1,500 Da was automatically eliminated to avoid the effects of SPA and other substances. Data were processed using the Biomarker Wizard, which identified 20 protein peaks with differences in intensity between the adenoma and control samples, including peaks at 8,565.84, 5,910.50, 8,694.51, 8,473.00, 4,355.27, 14,036.25, 4,480.67, 6,843.43, 3,324.01, 11,710.32, 4,099.27, 6,636.65, 12,866.68, 16,541.76, 13,754.56, 8,150.09, 14,976.34, 6,438.44, 2,489.73 and 7,976.21 Da. Colorectal adenoma samples were found to exhibit six low protein peaks compared with the control samples, where these peaks were detected at high intensity levels (Table I).

**Figure 1:**
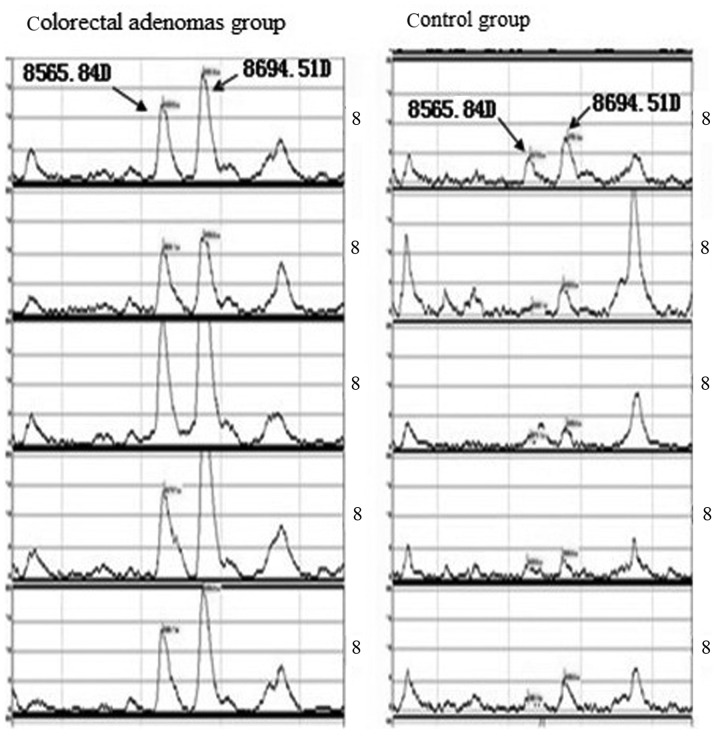
Expression of the 8,565.84 and 8,694.51 Da peaks in the colorectal adenoma and control groups.

**Figure 2:**
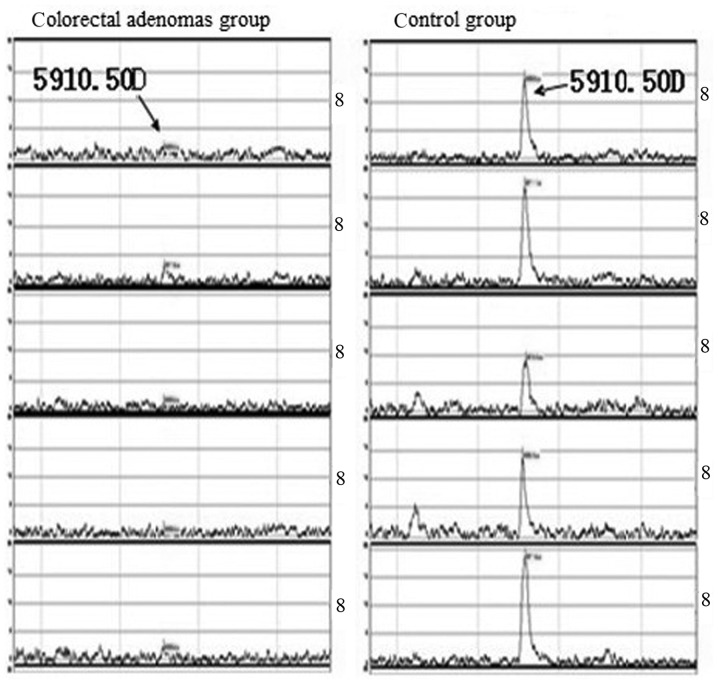
Expression of the 5,910.50 Da peak in the colorectal adenoma and control groups.

A statistically significant difference in peak intensity between the two groups was identified for three peaks, 8,565.84, 5,910.50 and 8,694.51 Da, in which 8,565.84 and 5,910.50 Da were expressed at high levels in colorectal adenoma patients and low levels in healthy subjects. By contrast, expression of the 5,910.50 Da peak was low in adenoma patients and high in normal control individuals (Table I and Figs. 1 and 2).

## Discussion

In recent years, the field of proteomics has developed rapidly. Clinical proteomics primarily focuses on the identification of protein disease biomarkers in body fluids, cells and tissues (11,12). SELDI-TOF-MS is a newly developed comparative proteomic technology and its clinical application is extensive (13–15). SELDI-TOF-MS has been used to detect small-molecule proteins that correlate closely with oncogenesis (16). A previous study by Grizzle *et al*(17) demonstrated that performing SELDI-TOF-MS using standardized instruments and controlled serum quality represents a promising approach for the detection of cancer during its early-stages.

Despite a number of studies on the use of SELDI-TOF-MS for the detection of colorectal cancer, the incidence of this cancer type has not been decreased significantly. The ‘adenoma-cancer sequence’ is a well-known hypothesis, which states that the detection of an adenoma followed by a resection may markedly reduce the incidence of colorectal cancer (18,19).

In the present study, SELDI-TOF-MS was performed in combination with the use of the WCX2 protein chip to analyze and compare the serum protein fingerprint of 50 colorectal adenoma patients with 50 healthy control individuals. Using the Biomarker Wizard, twenty protein peaks were identified to vary between the two groups, including seven peaks with a lower protein content in the adenoma group, of which, the 3 protein peaks, 8,565.84, 5,910.50 and 8,694.51 Da, were found show the greatest significant difference. Specifically, the 8,565.84 and 5,910.50 Da peaks were found to be expressed at high levels in the colorectal adenoma patients and at low levels in healthy subjects. By contrast, the expression of the 5,910.50 Da peak was low in the adenoma patients and high in the control participants. Based on these observations, we hypothesize that the expression of the 8,565.84 and 8,694.51 Da peaks may correlate with an oncogene, while the 5,910.50 Da peak may be associated with a tumor suppressor gene. However, further confirmation of these results must be acquired. The identification of the three protein peaks, 8,565.84, 5,910.50 and 8,694.51 Da, is of great significance and provides insight into the identification of novel markers specific to colorectal adenoma.

In comparison to a colonoscopy, SELDI-TOF-MS represents a minimally-invasive approach, requiring a venous blood sample only. SELDI-TOF-MS represents a promising technology for the early-stage diagnosis of colorectal adenoma and is associated with a number of advantages, including a high flux, no sample processing and the ability to identify small-molecule proteins. Future utilization of this technology to screen for colorectal adenoma in healthy populations is likely to significantly decrease the incidence of colorectal cancer.

## Figures and Tables

**Table I:** Protein fingerprint of serum protein in colorectal adenoma and control samples.

		Average intensity of protein peak (mean±SD)	

Protein peak (Da)	P-value	Colorectal adenoma	Control
5,910.50^*^	0.0002	2.53±0.34	14.55±1.65
8,565.84	0.0003	12.63±1.47	5.09±0.53
8,694.51	0.0003	17.02±1.99	7.11±0.78
8,473.00	0.0157	1.37±0.12	0.96±0.10
4,355.27	0.0157	4.66±0.49	2.96±0.33
14,036.25	0.0178	1.97±0.21	1.16±0.14
4,480.67^*^	0.0120	12.50±1.32	15.12±1.49
6,843.43	0.0129	3.76±0.38	2.39±0.27
3,324.01	0.0132	7.22±0.81	5.42±0.66
11,710.32	0.0132	6.05±0.71	4.03±0.52
4,099.27^*^	0.0174	5.48±0.49	8.09±0.77
6,636.65	0.0188	34.17±3.63	29.90±3.15
12,866.68	0.0223	0.87±0.07	0.54±0.04
16,541.76^*^	0.0223	0.87±0.06	1.25±0.09
13,754.56	0.0285	4.03±0.33	2.66±0.32
8,150.09^*^	0.0301	3.97±0.42	5.52±0.64
14,976.34	0.0301	0.33±0.05	0.03±0.04
6,438.44	0.0358	16.26±1.55	10.12±1.12
2,489.73	0.0317	6.18±0.78	4.97±0.52
7,976.21^*^	0.0317	11.43±1.36	16.96±1.75

* low protein peak relative to normal control.
